# Ophthalmic Hydrogel Contact Lens Material Containing Magnesium Oxide Nanoparticles and 3-(Trifluoromethyl)styrene for Biomedical Application

**DOI:** 10.3390/mi13111897

**Published:** 2022-11-02

**Authors:** Min-Jae Lee, Seon-Young Park, A-Young Sung

**Affiliations:** 1 Department of Optometry, Jeju Tourism University, Jeju 63063, Korea; 2 Department of Optometry & Vision Science, Daegu Catholic University, Gyeongsan 38430, Korea

**Keywords:** magnesium oxide nanoparticles, 3-(trifluoromethyl)styrene, hydrogel, ophthalmic lens material, oxygen permeability, tensile strength

## Abstract

This research was conducted for the synthesis and application of ophthalmic lens materials with improved oxygen permeability and durability. Polyvinylpyrrolidone (PVP), N-vinyl-2-pyrrolidone (NVP), 3-(trifluoromethyl)styrene (3-TFMSt), and magnesium oxide nanoparticles were used as additives for the basic combination of 2-hydroxyethyl methacrylate (HEMA). Additionally, the materials were copolymerized with ethylene glycol dimethacrylate (EGDMA) as the cross-linking agent and azobisisobutyronitrile (AIBN) as the initiator. The addition of magnesium oxide nanoparticles was found to increase the tensile strength from 0.0631 to 0.0842 kgf/mm^2^. Copolymerization with a small amount of 3-TFMSt of about 1% increased the tensile strength to 0.1506 kgf/mm^2^ and the oxygen permeability from 6.00 to 9.64 (cm^2^/s)∙(mLO_2_/mL·mmHg)∙10^−11^. The contact lens material produced using N-vinyl-2-pyrrolidone and magnesium oxide nanoparticles as additives satisfied the basic physical properties required for hydrogel contact lenses and is expected to be used usefully as a material for fabricating high-performance hydrogel lenses.

## 1. Introduction

As polymethyl methacrylate (PMMA) is hydrophobic and has excellent optical properties, hardness, and weather resistance, it can be used as a contact-lens material [1]. Additionally, it has excellent biocompatibility and non-toxicity, and it is not easily biodegradable [2]. However, PMMA has low water content, wettability, and oxygen permeability, so the hydrogel has been gradually used. Hydrogel is used in various fields, including medical care, life activity, material science, and engineering; in particular, it is widely used in contact lenses due to its excellent properties such as biocompatibility, optical properties, and mechanical strength [3]. As a typical example, the polymer consisting of poly (2-hydroxyethyl methacrylate) with excellent water content, mechanical properties, and biocompatibility is widely used as a material for hydrogel contact lenses [4]. Hydrogel lenses are used for various purposes, such as vision correction, ophthalmic treatment, and beauty, and are widely used due to the flexibility of their material and their excellent wearability. However, hydrogel contact lenses may cause problems such as corneal hypoxia and corneal edema due to low oxygen permeability [5]. Water content affects the wearability and fitting of contact lenses and is directly related to oxygen permeability and wettability [6,7]. Wettability is closely related to water content. If the wettability and oxygen permeability are low, corneal edema and the loss of corneal crust due to hypoxia occur, and dry eyes also occur due to changes in the tear layer [8]. As the sensation of dryness increases with the wearing of soft contact lenses, the choice of soft lens material is important. In addition, it has been reported that a silicone hydrogel lens with high oxygen permeability alleviates ophthalmic symptoms [9,10,11,12]. Silicone hydrogel lenses are manufactured by adding silicone to the existing hydrogel monomer, thereby providing high oxygen permeability. Therefore, higher safety can be secured compared to general hydrogel lenses [13]. Polydimethylsiloxane (PDMS)-based silicone hydrogel lenses containing silicone compounds have been developed and are used [14]. However, as the silicone monomer is hydrophobic, it does not mix well with hydrophilic compounds such as HEMA during polymerization, causing phase separation and the opacity of hydrogel lenses [15]. Similarly, various studies on the transparency of materials are continuing [16,17,18,19].

Hydrogel contact lenses, the medical devices for vision correction, can be directly worn on the human body. More than 60% of people use glasses or contact lenses, and contact lens users account for 20% of all users [20,21]. As the hydrogel contact lenses, which is an alternative to eyeglasses for vision correction, have a wide field of view and do not have cosmetic disadvantages that may occur while wearing eyeglasses, the number of users is increasing. In addition, contact lenses are used for cosmetic purposes in addition to their original purpose, i.e., vision correction, and the market is continuously growing [22,23]. Therefore, problems caused by wearing contact lenses and the methods of managing lenses should be further studied. In particular, with the recent progress in the endemicization of coronavirus diseases, hygiene management is urgently required to respond to bacteria and viruses for both individuals and society. As a result, consumers’ interest and demand for antibacterial and sterilizing products are rapidly increasing. Previous studies suggested that the main route of infection of COVID-19 is not only through the respiratory system but also through the eyes [24,25].

As the conjunctiva is directly exposed to pathogens and anatomically connected to the nasolacrimal duct, it can be transferred to the nasal cavity and nasopharyngeal mucosa through the lacrimal tract, causing respiratory infections [26,27]. Therefore, the eye exposed to the outside becomes a channel that acts as a direct receptor for bacteria and viruses. As soft lenses are in direct contact with the cornea, they act as a factor that interferes with the normal physiological function of the eyeball, causing side effects such as hypoxia, corneal edema, keratitis, erosion, corneal ulcer, and corneal neovascularization, in addition to wear-related subjective symptoms such as redness, dryness, and a feeling of irritation caused by foreign matters [28,29]. In addition, as they are in direct contact with the cornea, the side effects of infections such as corneal ulcers and keratitis may occur due to users’ negligent management and [30] inappropriate wearing [31,32]. Eye infections that can occur from wearing contact lenses are caused by bacteria, fungi, and viruses, and it is known that the bacterial keratitis caused by Staphylococcus aureus and Pseudomonas aeruginosa is the most common [33]. In the case of existing replacement lenses, despite regular cleaning, the deposits generated while wearing contact lenses caused side effects such as giant papillary conjunctivitis [34], keratitis [35,36], and corneal ulcer [37,38].

This study tried to improve various physical properties without using silicone material. Using styrene and magnesium nanoparticles, which were not previously used as contact lens materials; we tried to fabricate a new contact lens that contains the functions necessary for these days, such as antibacterial properties, and improves durability including oxygen permeability. In that context, this study improved the functionality of ophthalmic lens materials by adding various monomers to the basic monomer materials used as the materials for hydrogel hydrophilic ophthalmic lenses at different ratios, and measured and analyzed the photophysical properties of the prepared hydrophilic hydrogel lens.

## 2. Materials and Methods

### 2.1. Reagents and Materials

To prepare hydrogel lenses, for the main materials, i.e., 2-hydroxyethyl methacrylate (HEMA) and the initiator azobisisobutyronitrile (AIBN), the products from Junsei were used; and for the crosslinking agent, i.e, ethylene glycol dimethacrylate (EGDMA) and the additives for functional improvement, i.e, polyvinylpyrrolidone (PVP), N-vinyl-2-pyrrolidone (NVP), 3-(trifluoromethyl)styrene (3-TFMSt), and magnesium oxide nanoparticles, the products from Sigma-Aldrich (Saint Louis, MO, USA) were used.

### 2.2. Polymerization

HEMA, the basic material of hydrogel lenses, was used as the main material; AIBN was used as a thermal initiator; and EGDMA was used as a crosslinking agent. In addition, PVP and NVP, which are the additives; 3-TFMSt; and magnesium oxide nanoparticles were added to the main material at ratio of 5%: 1%; and 0.05 and 0.1%, respectively. After ultrasonication for about 30 min to disperse the nanoparticles, the mixture was stirred for about 6 h at room temperature using a stirrer (Vortex GENIE 2, Scientific Industries, Bohemia, NY, USA). In addition, to prepare lenses, the mixture was thermally polymerzied at 80 °C for 3 h using a cast-mold method. The prepared hydrogel samples were hydrated in sterile physiological saline for 24 h, and then physical and optical properties such as refractive index, light transmittance, and water content were evaluated. As for each sample used in the experiment, the experimental group without additives was named Ref; the group with 5% of PVP added to 0.05% of magnesium oxide nanoparticles was named M0.05P5. In addition, the group with 5% of PVP added to 0.1% of magnesium oxide nanoparticles was named M0.1P5; the group with 5% of NVP added was named M0.1N5; and the group with 1% of 3-TFMSt added to M0.1P5 and M0.1N5 was named M0.1P5S1 and M0.1N5S1, respectively. The mixing ratio of the hydrogel samples used in the experiment is presented in Table 1.

### 2.3. Analysis

The physical properties for confirming the usability of a contact lens include refractive index, light transmittance, water content, wettability, tensile strength, and oxygen transmittance. Among them, if the light transmittance is 88% or more in the visible light region, it is judged that there is no problem as a transparent lens. In the case of water content, 50% or less than 50% is classified as a low-water lens, and 50% or more is classified as a high-water content lens. In the case of wettability, if the contact angle is close to 0 degrees, it is classified as hydrophilic, and if it is close to 180 degrees, it is classified as hydrophobic. There is no minimum standard value for other physical properties. To confirm the practicality of the manufactured lens, in this study, the refractive index and the light transmittance were checked for optical properties; water content and wettability were checked for wearing comfort; tensile strength was checked for durability; and oxygen transmittance and antibacterial properties were checked for eye diseases. The thermogravimetric analyzer (TGA), the absorbance and extractable test for lens stability, and the atomic force microscope (AFM) for the surface analysis to confirm the surface characteristics were measured and analyzed.

The refractive index of the samples was measured 5 times for each lens based on the international standard ISO 18369-4:2006. The samples were hydrated in physiological saline solution for 24 h in an environment similar to the human eye and were measured using an ABBE Refractometer ((ATAGO DR-A1, Tokyo, Japan). For the water content, the gravimetric method was used, and the average value of the results, determined by experimenting 5 times for each lens, was presented. The spectral transmittance was measured using a spectral transmittance meter (Cary 60 UV-vis., Agilent, Santa Clara, CA, USA). To calculate the average, the UVB (280–315 nm), UVA (315–380 nm), and visible-light (380–780 nm) regions were measured 5 times each. The wettability was evaluated 5 times for each lens by measuring the contact angle using a contact-angle instrument (Contact Angle Instrument, DSA30, Kruss, GmbH, Hamburg, Germany), and the Sessile drop method was used. Shimadzu’s Universal Testing Machine AGS-X was used 5 times for each lens to examine the tensile strength of the lens, and an atomic force microscope (XE-100, Park Systems, Suwon, Korea) was used to measure the surface roughness of the lens. To measure the eluate, absorbance, pH change, and the presence of a potassium-permanganate-reducing substance were measured 5 times for each lens. The change in pH was considered to have no effect if the difference was less than 1.5 compared to the control, and in the case of the potassium-permanganate-reducing substance, if the difference was less than 2 mL compared to the control, it was considered to have no eluate. For the absorbance, a spectral transmittance meter (Cary 60 UV-vis., Agilent, Santa Clara, CA, USA) was used, and the absorbance was determined by measuring the highest wavelength 5 times each and calculating the average. The antibacterial properties against Staphylococcus aureus and Escherichia coli, which occur most frequently in the eye, were measured 5 times for each lens. For an incubator for bacterial culture, Daewon Science’s Shacking Incubator DS-210SL was used. In addition, the polarographic method was used to measure the oxygen permeability 5 times for each lens. For thermal stability analysis, weight changes according to decomposition temperature were analyzed using TGA Q500 of TA Instruments.

## 3. Results and Discussion

### 3.1. Thermal Properties

**Figure 1:**
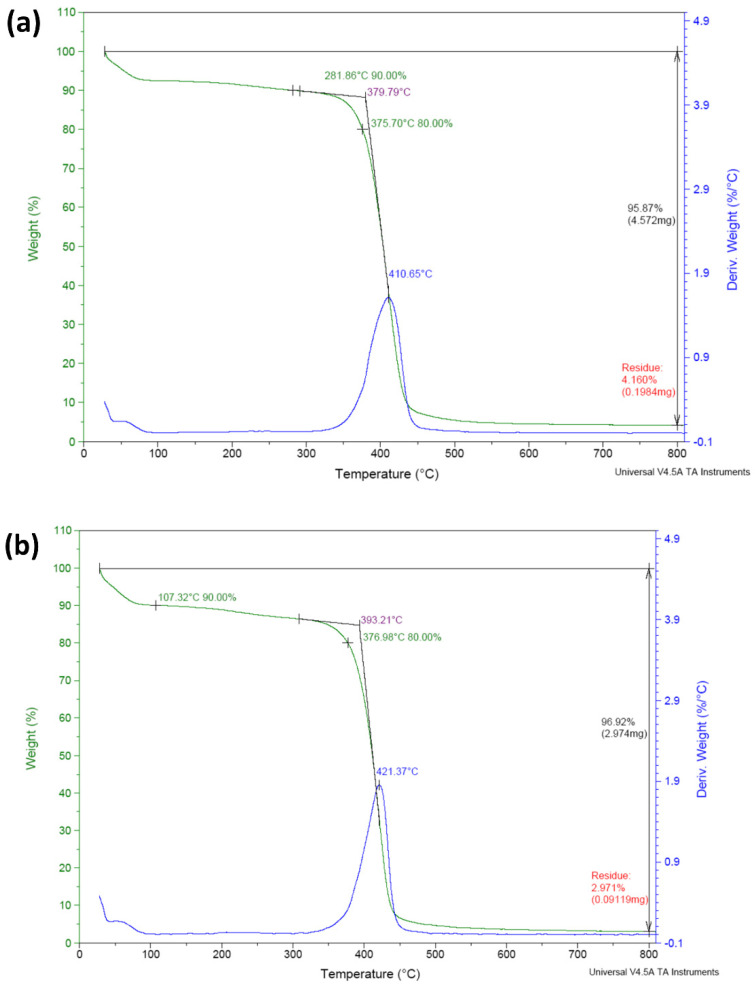
TGA analysis of (**a**) M0.1N5 sample and (**b**) M0.1N5S1 sample.

To evaluate the thermal stability according to the addition of 3-TFMSt on the contact lens samples, TGA analysis was performed. As presented in Figure 1a, the M0.1N5 sample exhibited three decomposition steps. In the first step, most samples exhibited a weight loss of 10% at 281.86 °C, which was judged to be related to the dehydration of the sample. In the second step, there was a weight loss of 20% at 375.70 °C. At this temperature, the decomposition and thermal decomposition of the molecular chain can begin. In the last step, the loss was confirmed at 410.65 °C due to the decomposition reaction of the main-chain and the deterioration of the polymer skeleton. In the case of the M0.1N5S1 sample, as shown in Figure 1b, in the first step, most samples exhibited a weight loss of 10% near 107.32 °C; in the second step, a weight loss of 20% was confirmed near 376.98 °C; and in the last step, a final loss of approximately 421.37 °C was confirmed. The M0.1N5S1 sample exhibited higher thermal stability than the M0.1N5 sample, showing a typical graph pattern in which a single material is thermally decomposed, so it was judged that uniform polymerization was achieved.

### 3.2. Physical Properties

#### 3.2.1. Refractive Index and Water Content

**Figure 2:**
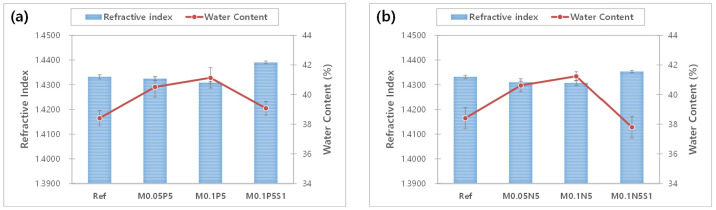
Change of refractive index and water content of samples. (**a**) PVP group; (**b**) NVP group.

The refractive index is a critical characteristic that indicates the optical properties of materials and affects the refractive power of contact lenses. Additionally, as it affects the strength, it is closely related to the wearing comfort. The results of the refractive index and the water content of each sample were compared with each other, as shown in Figure 2. According to the addition of magnesium oxide nanoparticles, PVP, and NVP to the Ref sample, the refractive index decreased slightly from 1.4331 to 1.4307. On the other hand, the water content improved from 38.41% to 41.24%. In the case of the M0.1P5S1 sample and the M0.1N5S1 sample with 3-TFMSt added, the refractive index increased to 1.4353~1.4390, and the water content decreased to 37.80~39.07% under the influence of styrene used as a material for the high refractive index. In the relation between the refractive index and the water content, the samples with high water content in general exhibited a low refractive index [39]. This study presented the same results as those of previous studies. Therefore, it could be seen that the addition of PVP and NVP increases the water content, and 3-TFMSt is a useful monomer for increasing the refractive index.

#### 3.2.2. Optical Transmittance

**Figure 3:**
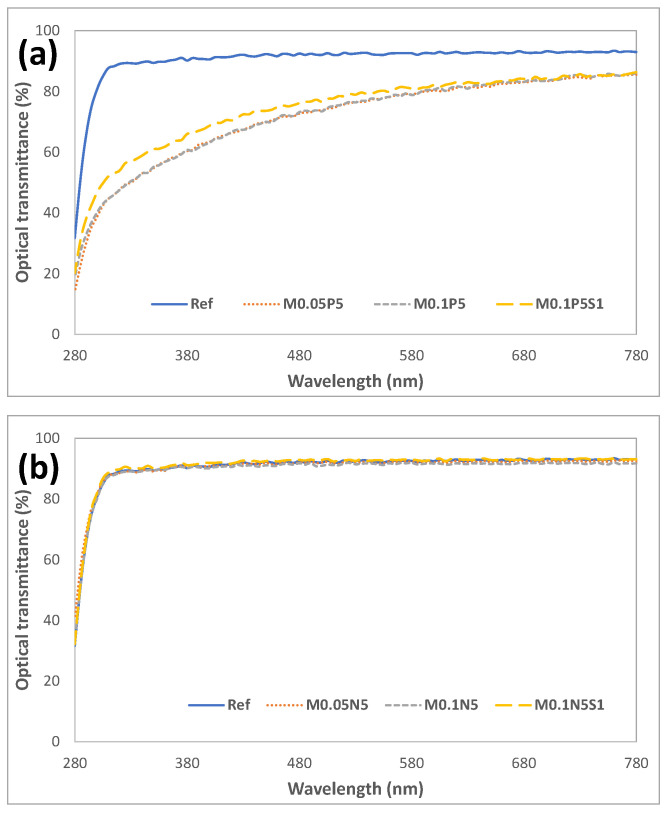
Optical transmittance of samples. (**a**) PVP group; (**b**) NVP group.

To examine the optical properties of each sample, the spectral transmittance was measured. The average visible light transmittance of the Ref was measured to be as significantly high as 92.09%, and it was 71.90% in UVB and 89.86% in UVA. As a result of measuring the average spectral transmittance of the M0.1N5S1 combination containing the additive, the visible light transmittance was 92.01%, and it was 72.49% in the UVB region and 88.79% in the UVA region. It was found that the addition of PVP, NVP, and magnesium oxide nanoparticles used in the experiment did not block UV rays but maintained visible light transmittance, indicating that the compatibility of the monomers was excellent. The light transmittance of each combination is presented in Figure 3. As the combination of monomers used in this experiment did not significantly change the physical properties and maintained the optical functions, the monomers are considered useful as additives for hydrophilic lens materials.

#### 3.2.3. Oxygen Permeability

**Figure 4:**
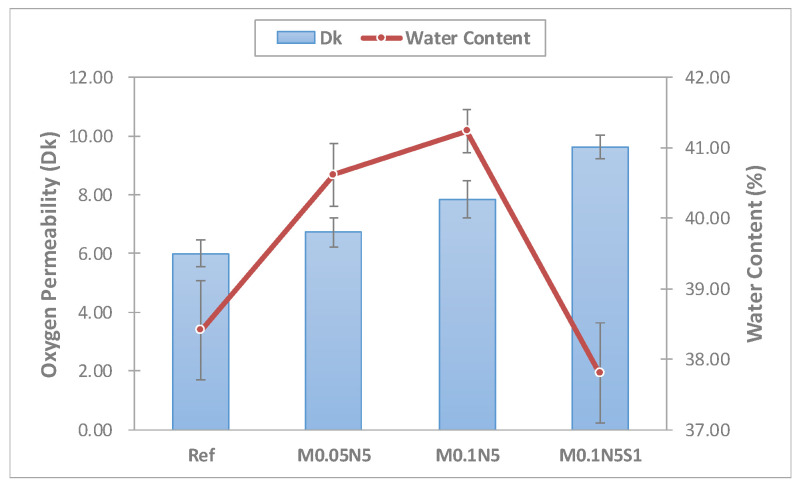
Oxygen permeability and water content of samples.

**Figure 5:**
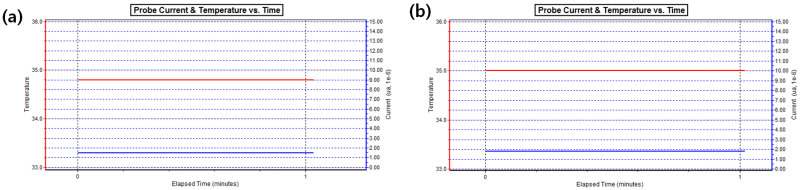
Probe current and temperature versus time in optic zone of (**a**) Ref sample and (**b**) M0.1N5S1 sample.

The oxygen permeability of the manufactured lens was measured. The Ref combination presented a value of 6.00 × 10^−11^(cm^2^/s)(mLO_2_/mL·mmHg); M0.05N5 and M0.1N5 with magnesium oxide nanoparticles added in proportions exhibited 6.72 × 10^−11^(cm^2^/s) (mLO_2_/mL·mmHg) and 7.84 × 10^−11^(cm^2^/s) (mLO_2_/mL·mmHg), respectively; and mmHg and M0.1N5S1 with 1% of 3-TFMSt added exhibited a value of 9.64 × 10^−11^(cm^2^/s)(mLO_2_/mL·mmHg), indicating that the addition of magnesium oxide nanoparticles and 3-TFMSt further increase the oxygen permeability of the lens. It was considered that the oxygen permeability increased by the increase in the water content according to the addition of magnesium oxide nanoparticles; the addition of 3-TFMSt actually decreased the moisture content, but the oxygen permeability increased. Based on these results, the addition of 3-TFMSt exhibited a trend opposite to that of the previous studies, suggesting that the oxygen transfer rate increased due to the water content. The relationship between the oxygen permeability and moisture content of each combination and the oxygen permeability current change graph are presented in Figure 4 and Figure 5, respectively.

#### 3.2.4. Tensile Strength

**Figure 6:**
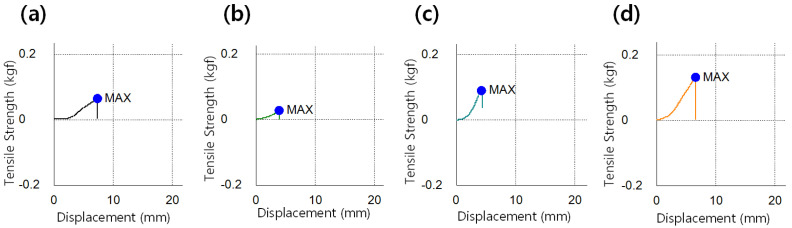
Tensile strength image of (**a**) Ref sample, (**b**) M0.05N5 sample, (**c**) M0.1N5 sample, and (**d**) M0.1N5S1 sample.

The strength of the prepared lens was determined through the tensile strength of the lens, and the results are presented in Figure 6. The tensile strength of the Ref was 0.1118 kgf/mm^2^, and in the samples M0.05P5, M0.05N5, M0.1P5, M0.1N5, and M0.05N5S1, it was 0.0561 kgf/mm^2^, 0.0631 kgf/mm^2^, 0.0810 kgf/mm^2^, 0.0842 kgf/mm^2^, 0.0986 kgf/mm^2^, and 0.1506 kgf/mm^2^, respectively. The magnitude of the tensile strength exhibited a tendency to decrease in the samples with the magnesium oxide nanoparticles, PVP, and NVP added, and it was improved by 34.7% in the sample containing 3-TFMSt. In general, the tensile strength tends to decrease as the water content increases. However, the results of this study indicated that the tensile strength was improved without a significant change in water content.

#### 3.2.5. Test for Absorbance and Extractables

**Figure 7:**
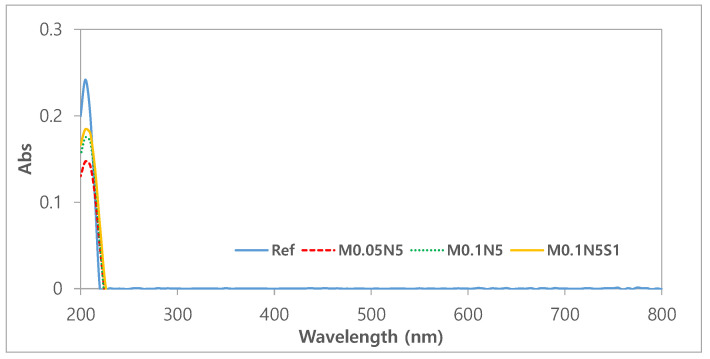
Absorbance of samples.

**Figure 8:**
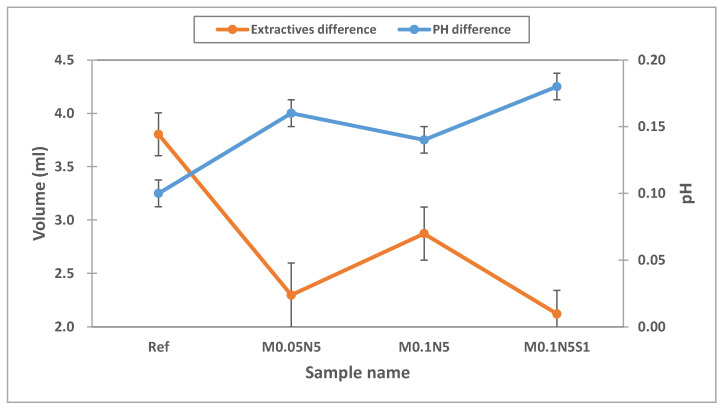
pH and extractables test of samples.

As contact lenses come into direct contact with the cornea, it is critical to ensure their stability [40]. To evaluate the polymerization stability of the prepared ophthalmic lenses, the absorbance, pH, and potassium-permanganate-reducing substances were tested [41]. To evaluate the elution of the NVP and 3-TFMSt used as additives and the nanoparticles used, the stability of Ref, M0.05N5, M0.1N5, and M0.1N5S1 was compared. As a result of measuring the absorbance of the hydrated solution, it was 0.242 in Ref; 0.147 in M0.05N5; 0.175 in M0.1N5; and 0.181 in M0.1N5S1. The absorbance varied according to the type of sample, but in conclusion, the absorbance of the copolymer was found to be decreased compared to that of Ref. The absorbance measurement results of each sample were presented in Figure 7. In the case of the pH difference experiment, it was 0.1 in Ref; 0.16 in M0.05N5; 0.14 in M0.1N5; and 0.18 in M0.1N5S1. As the value was below the standard value of 1.5 in all groups, it was judged that there was no eluate causing potential change. As a result of measuring the potassium-permanganate-reducing substance, the difference from the control group was 3.80 mL in Ref; 2.30 mL in M0.05N5; 2.87 mL in M0.1N5; and 2.12 mL inM0.1N5S1. Although the effect on the eluate was greatly reduced, it still exceeded the standard value of 2ml, indicating that further studies are required. As the polymerization state varies depending on the type of nanoparticles added, affecting the polymerization stability, it is judged that studies on the polymerization method, the polymerization conditions, and the selection of initiators and crosslinking agents should be continued in consideration of their mechanism. The eluate results according to pH and reducing substances were presented in Figure 8.

#### 3.2.6. Antimicrobial Test

**Figure 9:**
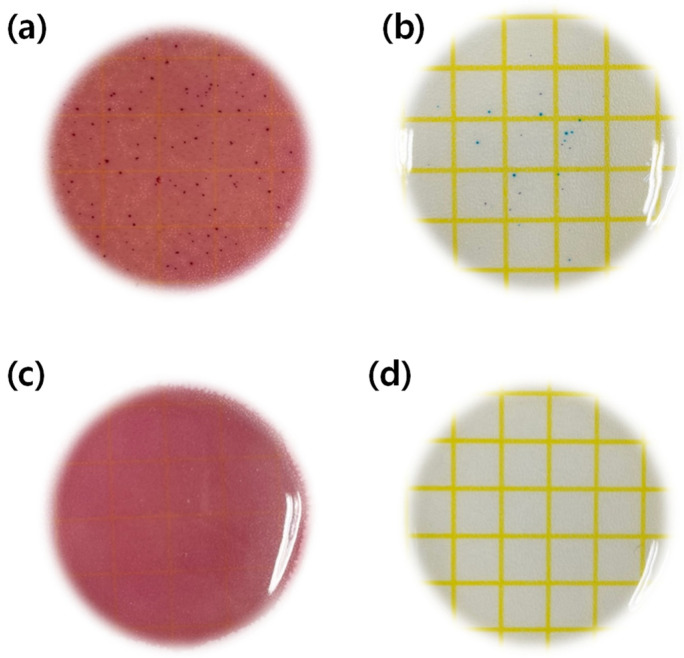
Antimicrobial test of samples. (**a**) Escherichia coli test of Ref sample, (**b**) Staphylococcus aureus test of Ref sample, (**c**) Escherichia coli test of M0.1N5S1 sample, (**d**) Staphylococcus aureus test of M0.1N5S1 sample.

To examine the antibacterial properties of nanoparticles, the antibacterial properties against Staphylococcus aureus and Escherichiacoli were experimented using the Ref, which did not contain the magnesium oxide nanoparticles, and the sample, which contained 0.1% of the magnesium oxide nanoparticles in HEMA, and the material of the hydrogel hydrophilic lenses, as the control (Figure 9a,b) and the experimental groups (Figure 9c,d), respectively. As a result, it was found that the distribution of bacteria appeared in contrast. The results of the antibacterial experiment using dry film medium for S. aureus and E. coli were presented in comparison in Figure 9. This study showed similar results to other studies on contact lenses containing silver nanoparticles, which are known to have antibacterial properties [42]. Therefore, judging from the results of this study, it would be possible to manufacture the hydrogel hydrophilic lenses with antibacterial function against Staphylococcus aureus and E. coli by using magnesium oxide nanoparticles as an additive.

### 3.3. Surface Property

#### 3.3.1. Wettability

**Figure 10:**
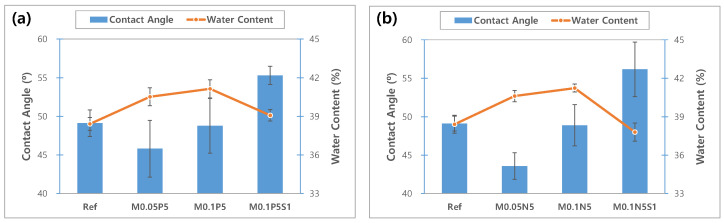
Change of contact angle and water content of samples. (**a**) PVP group; (**b**) NVP group.

**Figure 11:**
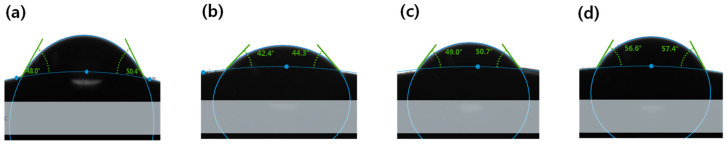
Contact angle image of (**a**) Ref sample, (**b**) M0.05N5 sample, (**c**) M0.1N5 sample, (**d**) M0.1N5S1 sample.

To evaluate the wettability of the sample, the contact angle was measured. The measured values for each sample, and the contact angle image, are presented in Figure 10 and Figure 11, respectively. In general, the material is classified into ‘hydrophobic’ and ‘hydrophilic’, based on a contact angle of 90°. As a result of measuring, the contact angle was 49.10° in Ref; 45.80° in M0.05P5; 43.58° in M0.05N5; 48.77° in M0.1P5; 48.89° in M0.1N5; 55.29° in M0.1P5S1; and 56.17° in M0.1N5S1, indicating that the combination of magnesium oxide nanoparticles with PVP and NVP improves the wettability of the lens. The wettability is closely related to the wearability of the lens, and as the wettability of the lens increases, dehydration is reduced when it is worn on the eye, and there is a tendency to produce fewer tear deposits [12]. The sample containing 3-TFMSt exhibited a value within 90° due to a slight increase in the contact angle compared to the sample without 3-TFMSt, indicating that it has good wettability. In general, the wettability increases in proportion to the water content, but in this experiment, on the contrary, the wettability and the water content exhibited an inverse relationship with each other. The wettability on the polymer surfaces is related to the polymer chain length, the molecular size, the slow motion of the long-chain molecules [43], and the surface roughness [44]. Therefore, it is judged that the contact angle varied due to the complex mechanism of these various causes.

#### 3.3.2. AFM Analyses

**Figure 12:**
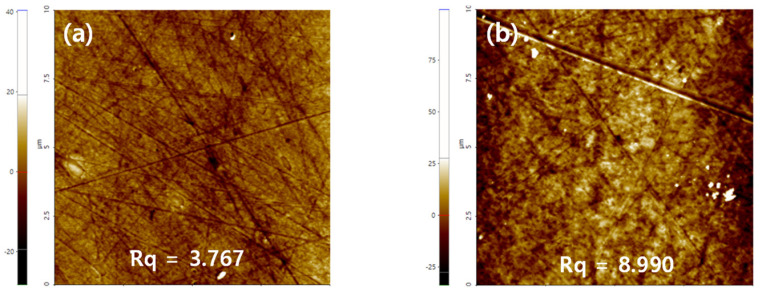
AFM analysis of (**a**) Ref sample and (**b**) M0.1N5S1 sample.

To analyze the roughness of the lens surface, the surface of the contact lens was analyzed using the AFM instrument. As a result, it was confirmed that the roughness of M0.1N5S1 increased compared with Ref. The increase in surface roughness due to the styrene among the various additives is considered to have an effect on the decrease in wettability [45]. The AFM image of each sample is presented in Figure 12.

## 4. Conclusions

This study analyzed the change in physical properties according to the addition of magnesium oxide nanoparticles, PVP, and NVP to hydrogel contact lenses, and the change in physical properties according to the addition of 3-TFMSt based on the aforementioned change. In the sample with magnesium oxide nanoparticles, PVP, and NVP added to the initial sample, the water content, wettability, oxygen permeability, and antibacterial properties increased, significantly improving the functionality of the contact lens. In addition, although a small amount of 3-TFMSt added increased the contact angle, the value was similar to that in the basic Ref, and the tensile strength increased by more than 1.34 times compared to the existing Ref sample while maintaining the physical properties of a hydrophilic hydrogel, indicating that 3-TFMSt is helpful in improving the functionality of hydrophilic contact lenses. The results of this experiment suggest that magnesium oxide nanoparticles and 3-TFMSt can be used in various ways as high-functional ophthalmic lens materials that satisfy the basic physical properties of contact lenses, and they can be used as lens materials with antibacterial properties in the era of the COVID-19 pandemic.

## Figures and Tables

**Table 1:** Percent composition of samples (unit: wt%).

Sample Name	HEMA	PVP	NVP	MO *	3-TFMSt	EGDMA	AIBN	Total
Ref	99.3	-	-	-	-	0.50	0.20	100
M0.05P5	94.53	4.73	-	0.05	-	0.50	0.20	100
M0.05N5	94.53	-	4.73	0.05	-	0.50	0.20	100
M0.1P5	94.48	4.72	-	0.10	-	0.50	0.20	100
M0.1N5	94.48	-	4.72	0.10	-	0.50	0.20	100
M0.1P5S1	93.55	4.68	-	0.10	0.98	0.50	0.20	100
M0.1N5S1	93.55	-	4.68	0.10	0.98	0.50	0.20	100

* MO: magnesium oxide nanoparticles.

## Data Availability

Not applicable.
